# Valid consent in the acute hospital setting: perspectives of patients and members of the public

**DOI:** 10.1007/s11845-024-03658-w

**Published:** 2024-04-05

**Authors:** Živa Kovic, Motheo Kobua, Mary Fogarty, Claire L. Donohoe, Michael E. Kelly, Gerard J. Fitzmaurice, Mella Fitzgerald, Paul Zambra, Una Geary, Marie E. Ward

**Affiliations:** 1 School of Medicine, Trinity College, The University of Dublin, Dublin 2, Ireland; 2 Quality and Safety Improvement Directorate, St James’s Hospital, Dublin 8, Ireland; 3 Gastrointestinal and General Surgery, St James’s Hospital, Dublin 8, Ireland; 4 Colorectal Surgery, St James’s Hospital, Dublin 8, Ireland; 5 Cardiothoracic Surgery, St James’s Hospital, Dublin 8, Ireland; 6 Centre for Innovative Human Systems, School of Psychology, Trinity College, The University of Dublin, Dublin 2, Ireland

**Keywords:** Capacity, Consent, Healthcare, Informed, Patients, Public, Voluntary

## Abstract

**Background:**

People who interact with healthcare services have an ethical and legal right to control their own lives, to make informed decisions, and to consent to what happens to them. For consent to be considered ethically and legally valid, three key criteria must be met: consent must be given voluntarily; people must be sufficiently informed of all options; and people should have capacity to make the decision to give or withhold their consent.

**Aim:**

This study set out to explore, through the use of surveys, the perspectives of patients and public in relation to consent.

**Method:**

Surveys were developed for patients and the public and administered paper based (patients) and through social media (public).

**Results:**

One hundred and forty surveys were posted to patients, with a 38% response rate; 104 responses were received from the public. Ninety-six percent of patients were satisfied that the decision they made was informed; 100% felt they had made a voluntary decision; 98% felt the clinician seemed knowledgeable about the procedure. What matters most to the public were being informed about the risks associated with the proposed procedure and being assured that whatever choice they make they will receive the best care possible.

**Conclusions:**

The results highlight interesting similarities and differences in relation to consent between members of the public thinking about a possible treatment, surgery, or procedure and those patients who have actually been through the process in the past 12 months. Recommendations have been developed on the basis of these findings to co-design improvements in consent practices.

**Supplementary Information:**

The online version contains supplementary material available at 10.1007/s11845-024-03658-w.

## Introduction

All people who interact with health and social care services have an ethical and legal right to control their own lives, to make informed decisions on matters that relate to them, and to decide and consent to what happens to them [1]. For consent to be considered ethical and legally valid, three key criteria must be met. Consent must be given voluntarily, patients must be sufficiently informed of all options, and patients must have capacity to make the specific decision at that time. The updated National Consent Policy [1] further defines the requirements about the extent and nature of the information that should be provided to patients while being invited to consent. This has been a vital focus of the new and incoming legislation including the Assisted Decision-Making (Capacity) Act [2]. This act will require a person’s capacity to be assumed and only to assess their capacity to make this specific decision if there is a concern. The act advocates for a human rights–based approach to supporting consent decision-making to be taken as opposed to personal or professional judgement on the part of the health and social care worker. It involves supporting a person’s needs in making a specific decision at a specific time.

In practice however in busy hospital environments, consent discussions can vary. Shoemaker et al. [3] identified that patients often sign consent forms without fully understanding the risks, benefits, and alternatives to treatment. In their study, as many as 58% patients were not able to teach-back their treatment plan and 54% were not aware of alternative treatment options. Akyüz and Erdem [4, p.529] observed that ‘33% had unanswered questions about the surgery’ and highlight that to understand their treatment plan, over half of patients wanted ‘healthcare professionals to avoid medical terminology’.

To ensure hospital patients are not only provided with sufficient information but are also able to understand it, Chia and Ekladious [5, p.892] argue for the use of language readily understandable to laypersons and checking with patients on their ‘understanding of messages’. Perni et al. [6] noted that the forms used to record consent were often not designed with patients’ needs in mind. The forms that they studied did not meet recommended readability index scores and used an average of 7.2 difficult words. They found that body site–specific forms had considerably better readability than general consent forms.

Convie et al. [7] in a systematic review of informed consent for surgery observed that patients and doctors felt that the transfer of knowledge was an important element of the consent process. They also found that communication skills by patients and clinicians were very important to the process and that sometimes, particularly in the public healthcare system, a patient’s desire to be seen as a ‘model patient’ impaired their ability to actively participate in the informed consent process [7].

Knight et al. [8, 9

In the National Inpatient Experience Survey [10] of patients admitted to acute public hospitals in Ireland, 39% of patients who responded stated that they did not have enough time to discuss their treatment with over one-third feeling they were not sufficiently involved in the decision-making process.

A recent systematic review has demonstrated a lack of studies using surveys that address all three key aspects of valid consent—informed, given with capacity, and voluntary [11]. After an extensive literature search of more than 10,000 potential survey studies, only sixteen survey scales were identified that assess any of the three domains of informed consent. None of the sixteen scales assessed all three domains of consent [11].

This study was carried out as part of a larger multi-disciplinary hospital wide co-design programme for consent practices using the HSE People’s Need Defining Change framework [12]. The aim of this study was to seek the perspectives of patients and members of the public in relation to all three key aspects of valid consent. The findings from this study would then feed into the wider co-design programme for consent practices across the hospital.

## Methods

### Study design and ethics

This study was carried out as part of a larger multi-disciplinary hospital wide longitudinal co-design programme for consent practices using the HSE People’s Need Defining Change framework [12]. The HSE framework proposes the following steps to change: identify, engage, define, design, and deliver. This study is part of the ‘engage’ phase of the change process and involves identifying the needs of patients and members of the public in relation to consent practices. Participants were given participant information leaflets (PILs) on the study and invited to participate. Participation was on a voluntary basis. No personal data was collected from participants. As both surveys were completed anonymously and voluntarily, informed consent was implied through the completion of the survey. All participants in the study were adults and in a position to give their own informed consent. The need for ethics approval was waived by the hospitals institutional review board (Ref: SJH R&I 7487). The study was carried out in accordance with the hospital guidelines and regulations for carrying out service evaluation and improvement projects.

### Survey development

As part of the wider improvement project, the hospital established a consent co-design project team. This team consisted of staff from the Quality and Safety Improvement Directorate and clinical staff from around the hospital. This team along with two medical students (ŽK, MK) designed the surveys for the patients and members of the public. Questions were formed around the three criteria for valid consent—being informed, voluntary, and given with capacity. All the questions were derived from a review of the literature and the national policy on consent. The full list of questions can be found in the Appendix 1 (patient) and Appendix 2 (members of the public).

On the patient survey, the first two questions were designed to ensure that patients only completed the survey if they had undergone a treatment, surgery, or procedure (hereafter referred to as treatment) by a hospital team within the previous 12 months and had signed a consent form. This was to ensure that their experience of consenting to treatment was relatively recent. The remaining questions consisted of thirteen statements where respondents replied with agree/disagree/not sure.

The public survey was broken into three sections with questions in each to find out about members of the publics’ views on each of the three criteria for valid consent (voluntary, informed, and capacity). Again, respondents were invited to reply with whether they agree/disagree/are not sure to a number of statements. After each of the three sets of statements, they were also asked to rate which statement was most important to them. This different format for the members of the public survey was chosen to assess their understanding of informed, voluntary, and with capacity to give consent, concentrating primarily on how they believe the hospital should ensure that all key criteria are considered. Surveys were tested on members of the co-design team before finalising. Qualtrics XM Solutions™ was used to create the online survey.

### Study participants and administration

#### Patients

It was originally intended to survey patients from three services in the hospital during outpatient day clinics. However due to COVID-19 clinic restrictions being in place, it was not possible to reach high enough numbers of survey participants at the first service. Therefore, it was decided that a postal survey targeting patients who had surgery in the previous 12 months would be more feasible. The clinical teams pre-screened patients for suitability, i.e. had surgery in the previous 12 months in the hospital or by the hospital team in another facility, and sent a list of the names of patients and addresses to the project team. The first batch of surveys were posted out to a sample of 60 patients from a second service (with three consultants) in May 2022. The second batch of surveys were sent out to a sample of 80 patients from a third service in June 2022.

The responses to these paper surveys were inputted manually into Qualtrics XM Solutions™ by two authors (ŽK, MK) and cross checked for any errors in data entry.

#### Members of the public

Information on the study and a link to complete the survey were sent out via the hospital’s social media accounts (Twitter, Facebook, and LinkedIn). The survey was sent out on 7th April 2022 and closed 2 weeks later. A reminder was sent out 7 days before the survey closed. The survey was open to all members of the public and no personal identifiable information was gathered. There was no way to determine if individuals completed the survey multiple times.

### Survey data analysis

Descriptive data analysis was carried out on the data supported by Qualtrics XM Solutions™. Percentage responses are calculated out of the total number of completed responses to each question. The Checklist for Reporting Of Survey Studies (CROSS) quality appraisal tool for carrying out web-based and non-web-based surveys [13] was used and can be found in the Supplementary files.

## Results

### Survey response rates

One hundred and forty surveys were posted out to patients and 53 completed surveys were received back (response rate 38%). One person reported that they did not have surgery in the last year so this was excluded, giving the final number of 52 participants. From the public survey distributed by social media in April 2022 by the hospital communications office, 126 responses were received, of which 104 were completed all the way through. Some participants did not complete all questions. Percentage responses are calculated out of the total number of responses to each question.

The results are presented here under the three pillars.

### Results of patient survey

#### Informed

The results of the informed section can be seen in Table 1. From this table, we can see that 96% of patients reported that they were satisfied that the decision they made to consent was an informed decision, 92% felt their clinician explained the treatment, and 90% felt their clinician gave them information about the benefits. Patients reported being dissatisfied with knowing that the treatment might be performed by a doctor other than the doctor taking their consent (29%); the information given about a number of treatment options including alternatives to the proposed treatment (26%); and that they were given written information (20%). The results are presented in Table 1 in percentages of responses and absolute numbers.

**Table 1:** Patients’ responses in relation to the questions about being informed in relation to their procedure/surgery

	Agree	Disagree	Not sure
I am satisfied that the decision I made to consent to the planned surgery/procedure was an informed decision	96% (50)	0	4% (2)
Prior to giving my consent, the doctor/nurse described and explained what was involved with the planned surgery/procedure	92% (48)	2% (1)	6% (3)
Prior to giving my consent, the doctor/nurse informed me about the benefits of the planned surgery/procedure	90% (47)	8% (4)	2% (1)
Prior to giving my consent, I was given all the information I needed to make a decision	88% (46)	4% (2)	8% (4)
Prior to giving my consent, the doctor/nurse informed me about the risks and/or possible complications associated with the planned surgery/procedure	87% (45)	8% (4)	6% (3)
Prior to giving my consent, the doctor/nurse informed me about a number of treatment options that were available to me including alternatives to the surgery/procedure that I had	58% (29)	26% (13)	16% (8)
Prior to my surgery/procedure, I was aware that the surgery/procedure might be performed by a doctor other than the doctor taking my consent	56% (29)	29% (15)	15% (8)
Prior to giving my consent, I was provided with written information about the planned surgery/procedure	55% (28)	20% (10)	25% (13)

#### Voluntary

In relation to whether patients felt their consent was given on a voluntary basis, 100% of respondents to this question agreed that it was. Seventy-seven percent of respondents reported that they were made aware that they could change their mind at any time. The results are presented in Table 2.

**Table 2:** Patients’ responses in relation to the questions about whether they perceived their consent to be voluntary in relation to their procedure/surgery

	Agree	Disagree	Not sure
I am satisfied that the decision I made to consent to the planned surgery/procedure was made freely, i.e. voluntarily	100% (52)	0	0
Prior to my surgery/procedure, I was made aware that I could change my mind (withdraw consent) at any time	77% (40)	15% (8)	8% (4)

#### Capacity

In order to ascertain patients’ views on capacity, it was felt important to ask them questions about the conditions which would support their understanding and ability to make decisions, for example, a knowledgeable clinician who took time with them and conveyed information in a manner that could be understood. Thus, for the section on capacity, we focused on three questions: whether the person felt the clinician was knowledgeable, whether information was conveyed to the patient in a manner they could understand, and if they were given enough time to make their decision. The results of these three questions can been seen in Table 3.

**Table 3:** Patients’ responses in relation to the questions related to capacity to make decisions in relation to their procedure/surgery

	Agree	Disagree	Not sure
The doctor/nurse who discussed the planned surgery/procedure with me and sought my consent seemed knowledgeable and well informed about the planned surgery/procedure	98% (51)	2% (1)	0
The doctor/nurse who discussed the planned surgery/procedure with me and sought my consent provided me with information in a way that I could understand	96% (50)	4% (2)	0
Prior to giving my consent, I was given adequate time to make a decision about having the surgery/procedure offered	90% (47)	6% (3)	4% (2)

#### Patients’ additional remarks and observations

Twenty additional comments were received, not all of which related to the consent process. Those that did are noted here:

- *I would have [liked to have] been informed about the after surgery. I had to ask what I was able to do and I had to consult a friend (doctor) to have advice for my health after the surgery!*
- *The doctor told me all, and she drew me a short plan of where she was going to remove the tumour. It went well and I am feeling well now.*
- *Would like to have been given more information as to alternatives to surgery e.g. my tumour had completely shrunk after radiotherapy/chemotherapy treatment. In retrospect the recovery has been so horrendous I feel I might not have gone for the surgery had alternatives been better discussed with me*
- *In relation to Q9: I knew the consultant who had been informing me was definitely the person doing the surgery*
- *Prof explained everything to me prior to the operation and treatment and I was satisfied to give my consent. I was treated very well by Prof and his team and I am very grateful.*

### Results of members of the public survey

#### Informed

The results from the public survey are presented here. What is different about this survey is that members of the public were also asked to respond yes/no to whether they believed certain information was important for them to receive. Members of the public were also asked to rate which information was most important to them. Under the criteria of ‘informed’, the top two statements that most members of the public (99.2%) agreed they would need details on were the potential impact of the planned treatment on their life and information on the benefits of the proposed treatment or procedure. However, what members of the public deemed most important was information about the risks and complications associated with the proposed treatment (33% agreed it was the most important information). The results of this part of the survey are found in Table 4.

**Table 4:** Members of the public responses in relation to the questions about being informed in relation to their treatment

In order for me to give my consent (agree to) or decline a medical treatment, surgery, or medical procedure recommended to me by a doctor or nurse or health and social care professional (medical team), I would expect to be provided with:		
Please pick one answer for each question	Yes (out of 123)	What information is most NB to me (out of 123)
1. Information about the risks and complications associated with the proposed treatment/procedure	119 (96.7%)	41 (33.33%)
2. Details on the potential impact of the planned treatment/procedure on my life	122 (99.2%)	27 (22%)
3. Information on the benefits of the proposed treatment or procedure	122 (99.2%)	19 (15.5%)
4. Information on the alternative treatments for me including no treatment at all	116 (94.3%)	14 (11.4%)
5. A description of the planned treatment/procedure	121 (98.4%)	11 (9%)
6. No information, I would like the medical team to proceed with the treatment they recommend	6 (4.9%	6 (5%)
7. Details on how to deal with any complications/side effects should they arise	109 (88.6%)	4 (3%)

#### Voluntary

Under the voluntary pillar, the responses that most members of the public affirmed were that they were assured that whatever choice they made they would receive the best care possible (85%) and that they were reassured that they could change their mind at any stage without their decision having any negative impact on their future/ongoing treatment and care (84%). The statement with which most members of the public agreed was most important was that they were assured that whatever choice they made they would receive the best care possible (38%). The next most important statement for respondents was that they would have other options explained to them in detail by the medical team providing the planned treatment (32%). The results of this part of the survey are found in Table 5.

**Table 5:** Members of the public responses in relation to the questions about whether they perceived their consent to be voluntary in relation to their procedure/surgery

In order for me to feel that I was/am completely free to give my consent (agree to) or decline a treatment, a surgery, or a medical procedure recommended to me by the medical team, I would require/need the following:		
Please tick one answer for each question	Yes (out of 123)	What is most NB to me? (out of 123)
1. Be assured that whatever choice I make I will receive the best care possible	105 (85%)	47 (38%)
2. Have other options explained to me in detail by the medical team providing the planned treatment/surgery/procedure	98 (80%)	32 (26%)
3. Be provided with adequate time to reflect on and discuss my options with my family/friends/carers	98 (80%)	12 (10%)
4. Be reassured that I can change my mind at any stage without my decision having any negative impact on my future/ongoing treatment and care	103 (84%)	10 (8%)
5. Be provided with the opportunity to talk to medical teams in the same fields/specialities, other than the team who have suggested the treatment/surgery/procedure to me	65 (53%)	6 (5%)
6. Have the opportunity to seek a second opinion in another hospital	74 (60%)	3 (2%)

#### Capacity

In relation to capacity, the item that most members of the public agreed was necessary corresponded with the statement identified as being most important—that the medical team would assist them in making a decision by providing them with additional information in a way they would understand (80% said yes and 50% agreed it was the most important—this was the highest agreement reached on any item in the public survey). The results of this part of the survey are found in Table 6.

**Table 6:** Members of the public responses in relation to the questions related to capacity to make decisions in relation to their procedure/surgery

If at the time I did not feel in a position to make a decision on whether to consent (agree to) or decline the treatment/procedure being offered to me, I believe the following should be undertaken:		
Please tick one answer for each question	Yes (out of 123)	What is most NB to me? (out of 123)
1. The medical team assists me in making a decision by providing me with additional information in a way I can understand	98 (80%)	62 (50%)
2. The medical team contacts my ‘next-of-kin’ or ‘contact person’ and asks them to make a decision about agreeing or declining for me	47 (38%)	20 (16%)
3. The medical team makes the decision for me	25 (20%)	8 (6.5%)
4. The medical team assists me in making a decision by providing me with additional time to make a decision	92 (75%)	7 (6%)
5. The medical team asks or checks to see if I have completed an advance directive (living will)	74 (60%)	7 (6%)

Table 7 presents a summary of the results with members of the public responses juxtaposed with patients’ responses.

**Table 7:** Summary of responses from members of the public and patients

	Public What is most important to me	%	Patients’ experience of consent process	%
Informed	Information about the risks and complications associated with the proposed treatment/procedure	(96.7% noted it was important; 33% noted it was most important)	Satisfied the decision they made was informed They would have liked more information about alternatives and more written information	(96% satisfied) (58% satisfied with information on alternatives) (55% satisfied with written information)
	Details on the potential impact of the planned treatment/procedure on my life	(99.2% noted it was important; 22% noted it was most important)		
Voluntary	Be assured that whatever choice I make I will receive the best care possible Have other options (alternatives) explained to me in detail by the medical team providing the planned treatment/surgery/procedure	(85% noted this was important; 38% noted it was most important) (80% noted this was important; 26% noted it was most important)	Satisfied they had made a voluntary decision Reported being given enough information on alternatives Reported that prior to their surgery/procedure they were made aware that they could change their mind (withdraw consent) at any time	(100%) (58%) (56%)
Capacity	The medical team assists me in making a decision by providing me with additional information in a way I can understand	(80% noted it was important; 50% noted it was most important)	Patients who completed the survey felt that the doctor/nurse who discussed the planned surgery/procedure with them and sought their consent seemed knowledgeable and well informed about the planned surgery/procedure Reported they were provided with information in a way that they could understand to make their decision	(Knowledgeable and well informed about the planned surgery/procedure 98%) (Information in a way that they could understand to make their decision 96%)

#### Members of the public additional remarks and observations

Twelve additional comments were received, not all of which related to the consent process. Those that did are noted here:

1. *Consent should be made by a patient who is in a position to reflect on discussion and has time to ask questions if the procedure is not an emergency. Arriving with a form to be signed within 2 min of an initial discussion may not be in the best interest of a nervous or often overwhelmed patient*
2. *There was no mention of the Assisted Decision Making supports that will come into being in June this year. Will and preference should be used rather than contacting ‘next of kin’ or close contacts!!!*
3. *This is very important work, I delighted to see somebody is working on these issues*
4. *Where a patient consents to one procedure but during the course of this procedure another procedure is required the patient should be given the opportunity to consent to the new procedure, except in an emergency or lifesaving situation*
5. *Patients’ advocate involvement—critical in serious decisions*
6. *My body my choice and should not be tormented for my decision [Re Covid vaccine]*

## Discussion

The results of this study highlight some interesting similarities and differences between members of the public thinking about a possible treatment and those patients who have actually been through the process in the past 12 months. In relation to making informed decisions, members of the public agreed that the most important information to be provided with before treatment would be information about the risks and complications associated with the proposed treatment. However, for patients who had been through the process more recently, while they felt they had been given all the information they needed to make a decision (88%) including information on risks and benefits (87%), some patients noted some aspects of their experience that could be improved. These included receiving written patient information material (PIM) in advance, being informed that the clinician obtaining consent might not be the same as the clinician performing the treatment, and receiving information on a number of treatment options that were available to them including alternatives to the treatment that they had. Members of the public would also like more details on the potential impact of the planned treatment on their life (99.2% noted this was important).

In relation to making voluntary decisions, 100% of patients felt they had made a voluntary decision. Being able to make a voluntary decision was also important to members of the public—with 85% noting that it was important that they were assured that whatever choice they made they would receive the best care possible. Thirty-eight percent of the public respondents agreed this was most important dimension in relation to questions under the voluntary criteria.

In relation to the capacity questions, the public reported that what was most important to them was that the medical team would assist them in making a decision by providing them with additional information in a way they can understand (80% agreed it was important; 50% agreed it was most important). After this, the most important thing for members of the public would be that the medical team contacted their ‘next-of-kin’ or ‘contact person’ and asked them for help in making a decision (38% noted it was important; 16% noted it was most important). Even though this term ‘next-of-kin’ is no longer a valid legal term, this was used in the survey as it is still commonly used by both staff and patients across the hospital.

It must be noted however that all of our patient respondents were people who had surgery in the last 12 months and this may be a factor in our mostly positive results from patients and hence a limitation of our study. Lattig et al. [14] found that surgeons believe that ‘patients consistently had higher expectations’ after the pre-operative discussion than the surgeons did. MacMahon et al. [15] found that more than two-thirds of patients had significantly higher expectations than their surgeons following discussions. For surgeons, responsibility for decision-making is very important [16]. In their meta-synthesis of surgeons’ perspectives, Orri et al. found that although surgeons took personal responsibility for choosing to operate on a patient, the need to share this responsibility with patients was also clearly expressed by surgeons [16]. Surgeons felt their responsibility as a personal commitment to deal with any complications that might arise during the surgery. A mutual commitment through post-operative care was thus actively sought by surgeons during the pre-operative encounters and consent conversations. Consent to surgery is taken in written from and thus explicitly given. Not all forms of consent in health and social care settings are given in written format.

A human rights–based approach to consent involves health and social care professionals engaging in shared decision-making (SDM) with patients [17]. SDM is a joint process in which a healthcare professional works together with a person to reach a decision about care, and involves choosing tests and treatments based both on evidence and on the person's individual preferences, beliefs and values.

In order to facilitate effective SDM, patients need to be informed of the risks, benefits, and possible consequences associated with the different treatment options available to them through clear and accessible discussions [17]. Engaging in real SDM however can be challenging for both clinicians and patients. Barriers to SDM can include clinician understanding of what is important to patients (e.g. the burden of treatment, treatment focusing on comfort or on living as long as possible, or side effects). Tools that support ascertaining patients’ preferences in medical decision-making are often limited to supporting patients to express preferences about a fixed set of treatment options [18]. Rietjens et al. [18] argue that what is needed is a radical new type of conversation tool, one that invites clinicians, patients, and their wider support system to engage in meaningful conversations about the ‘lived experience’ of illness while appreciating the different ways in which patients experience and navigate their illness. Such tools would also support more informed consent conversations.

This study has led to the development of recommendations for improvement in the hospital. One key area for improvement arising from this study is in relation to supporting consent conversations through providing patients as early as possible with PIMs about their treatment and the risks and benefits of that treatment. PIMs can provide a useful support tool for both patients and staff to facilitate consent conversations if they are tailored to the individual through discussions with their clinician, as the national consent policy notes ‘the risks that an individual person considers significant and relevant to their decision-making, can only be determined by discussion with them and by considering their will and preferences… Factors such as a persons’ occupation, lifestyle or culture may, for example, influence those risks that the person considers to be significant or particularly undesirable’ [1, p.22]. PIMs need to address the risks and benefits of all treatment options including none, and as found in this study and others, patients want information about the impact of the treatment options on their daily life in the short and long term [19]. It is also recommended that once patients have PIMs, they need ‘time out’ to review them and be able to come back to the conversation with their clinician with any questions relating to the information in the PIMs [19]. The hospital is currently exploring information and communication technology solutions that would support the tailoring of PIMs by clinicians for individual patients and their families and iterative interactive communication between clinicians and patients.

A second key area for improvement that the survey results have prompted is in relation to the tools used within the hospital to support the recording of consent conversations. The hospital has an electronic health record (EHR) and this may be used to support this process. In the first instance however, the paper-based form currently used to capture the consent conversation will be redesigned based on feedback from these surveys to better support a SDM process [17] in relation to the treatment. This form will then be embedded into the EHR system.

Other recommendations arising from the results of this study are to explore the perspectives of junior doctors (defined as those doctors who have graduated from medical school in the last year (interns) and two to 3 years (senior house officers (SHOs)) [20] and nursing staff in relation to their needs and experiences of consent practices. An information and awareness campaign for patients and their families about their role in consent conversations and SDM and the hospital being a teaching and research active hospital is also being undertaken. Empowering patients to engage in consent conversations and SDM is also essential to improving health literacy, which is a key objective of the World Health Organization [21]. Improving health literacy may also give public patients the skills to move beyond feeling the need to be a ‘model patient’ [7] and instead to be more actively engaged in understanding the risks and benefits of all their treatment options including none. When patients are more actively engaged in all aspects of their care, this can lead to greater improvements in quality and safety of care [22].

### Strengths and limitations

Members of the public were recruited through our hospital’s social media accounts which may have introduced bias as people who follow the hospital on social media may have had themselves, or members of their family or friends, a predominately positive experience of their care in the hospital. Questions were phrased in such a manner however to elicit what was most important to them. Also, the free text comments reflected both positive and negative experiences. Future research should try to engage more diverse representation from both patient and public groups. Future research also needs to focus on consent in other aspects of health and social care, particularly those for which currently explicit consent is not always sought, e.g. taking bloods and moving and handling patients.

This study did not explore fully the issue of capacity where there was any question that the patient did not have the ability to make this particular decision in this moment. Capacity in this study was related to a patient being given enough information and in a way they could easily understand, which enabled them to make their own decision.

## Conclusions

This study explored the perspectives of patients and members of the public in relation to three aspects of ethically and legally valid consent. The results from this study will support the co-design of new consent practices and processes within the hospital and can be used to inform improvements in the wider health system.

### Electronic Supplementary Material

Below is the link to the electronic supplementary material.

Supplementary file1 (DOCX 20 KB)

## Data Availability

All data generated or analysed during this study are included in this published article (and its supplementary information files).

## Appendix 2 Members of the public survey

Which of the above would be most important to you? Please tick 1, 2, 3, 4, 5, 6, 7, 8

Which of the above would be most important to you? Please tick 1, 2, 3, 4, 5, 6, 7

Which of the above would be most important to you? Please tick 1, 2, 3, 4, 5, 6, 7

Any other comments

Thank you very much for taking the time to complete this survey.
